# Characterization of the bark storage protein gene (*JcBSP*) family in the perennial woody plant *Jatropha curcas* and the function of *JcBSP1* in *Arabidopsis thaliana*

**DOI:** 10.7717/peerj.12938

**Published:** 2022-02-08

**Authors:** Ming-Jun Zhang, Qiantang Fu, Mao-Sheng Chen, Huiying He, Mingyong Tang, Jun Ni, Yan-Bin Tao, Zeng-Fu Xu

**Affiliations:** 1 School of Life Sciences, University of Science and Technology of China, Hefei, Anhui, China; 2 CAS Key Laboratory of Tropical Plant Resources and Sustainable Use, Xishuangbanna Tropical Botanical Garden, The Innovative Academy of Seed Design, Chinese Academy of Sciences, Menglun, Mengla, Yunnan, China; 3 State Key Laboratory for Conservation and Utilization of Subtropical Agro-Bioresources, College of Forestry, Guangxi University, Nanning, Guangxi, China

**Keywords:** *Jatropha curcas*, *JcBSP* gene family, Seasonal nitrogen cycling, Nitrogen induction, Overexpression, *Arabidopsis thaliana*

## Abstract

**Background:**

Bark storage protein (BSP) plays an important role in seasonal nitrogen cycling in perennial deciduous trees. However, there is no report on the function of BSP in the perennial woody oil plant *Jatropha curcas*.

**Methods:**

In this study, we identified six members of *JcBSP* gene family in *J. curcas* genome. The patterns, seasonal changes, and responses to nitrogen treatment in gene expression of *JcBSP*s were detected by quantitative reverse transcription-polymerase chain reaction (qRT-PCR). Overexpression of *JcBSP1* in transgenic *Arabidopsis thaliana* was driven by a constitutive cauliflower mosaic virus (CaMV) *35S* RNA promoter.

**Results:**

*JcBSP* members were found to be expressed in various tissues, except seeds. The seasonal changes in the total protein concentration and *JcBSP1* expression in the stems of *J. curcas* were positively correlated, as both increased in autumn and winter and decreased in spring and summer. In addition, the *JcBSP1* expression in *J. curcas* seedlings treated with different concentrations of an NH_4_NO_3_ solution was positively correlated with the NH_4_NO_3_ concentration and application duration. Furthermore, *JcBSP1* overexpression in *Arabidopsis* resulted in a phenotype of enlarged rosette leaves, flowers, and seeds, and significantly increased the seed weight and yield in transgenic plants.

## Introduction

Seasonal nitrogen cycling (SNC) is important in deciduous perennials to ensure the sufficient use of nitrogen resources. It is also a decisive factor for plant fitness in perennial (May & Killingbeck, 1992). The process of SNC involves the degradation of proteins when leaves shed in autumn, the transportation of the released amino acids to the perennial tissues (bark and wood) to synthesize storage proteins, and then used for the growth of new stems and leaves in spring (Babst & Coleman, 2018; Wildhagen et al., 2010). When the deciduous perennial trees overwinter, nitrogen is transported from senescing leaves to perennial tissues for storage (Ryan & Bormann, 1982). For example, before poplar leaf senescence, protein is hydrolyzed and transported to stems and roots in the form of amino acids, which results in approximately 90% of the nitrogen being removed from the leaves (Chapin & Kedrowski, 1983; Pregitzer et al., 1990). Through longitudinal section observation of *Populus* stems in winter and summer, it has been found that the phloem parenchyma cells and xylem ray cells contained only a large central vacuole in summer, while the central vacuole in these cells was replaced by many small protein storage vacuoles in winter (Clausen & Apel, 1991; Cleve & Apel, 1993; Cleve, Clausen & Sauter, 1988; Cooke & Weih, 2005; Sauter, Cleve & Wellenkamp, 1989; Sauter & Cleve, 1992; Wetzel, Demmers & Greenwood, 1989a). This protein is a kind of vegetative storage protein (VSP) and is a designated bark storage protein (BSP) (Cooke & Weih, 2005). It is an important form of nitrogen storage for perennial woody plants in winter.

Previous studies have shown that the poplar *BSP* is composed of a multigene family, including three subfamilies: *BSP*, *wound*-*inducible 4* (*WIN4*), and *poplar nitrogen*-*inducible 288* (*PNI288*) (Coleman, Chen & Fuchigami, 1992; Wildhagen et al., 2010). All three subfamily genes can respond to nitrogen induction (Coleman, Baíiados & Chen, 1994; Lawrence et al., 2001; Lawrence et al., 1997), but only *BSP* has been found to exhibit consistent seasonal expression changes with the total protein concentration in bark. The expression of *BSP* was increased in winter, whereas the expression of *WIN4* and *PNI288* increased only in spring (Wildhagen et al., 2010), and *BSP* has been reported to respond to short light duration and low temperature induction (Black et al., 2001; Cleve & Apel, 1993; Coleman et al., 1991; Coleman et al., 1993; Lawrence et al., 2001; Wildhagen, Bilela & Rennenberg, 2013). Therefore, only *BSP* is directly related to nitrogen storage during plant dormancy in winter among these three subfamilies.

*Jatropha curcas* is a perennial woody oil plant of the Euphorbiaceae family. It has received widespread attention because its seed oil is recognized as a promising feedstock for biodiesel production (Divakara et al., 2010; Kamel et al., 2018; Makkar & Becker, 2009; Mazumdar et al., 2018; Mohibbeazam, Waris & Nahar, 2005; Pandeya et al. 2012; Pramanik, 2003; Vaknin et al., 2018; Yi et al., 2014). Although *J. curcas* grows in tropical and subtropical regions, it is also a deciduous tree. Adult *J. curcas* begins to defoliate in autumn and stays dormant in winter until the next spring, when it enters the growing season. In this study, to identify *J. curcas BSP* (*JcBSP*) genes that may be involved in seasonal nitrogen cycling, we examined the expression of *JcBSP* family members in response to seasonal changes and nitrogen induction and found that the expression of *JcBSP1* was positively correlated with the total protein concentration in the stems during seasonal changes and with the exogenous nitrogen application. To further determine the roles of *JcBSP1* in plant growth and development, we characterized phenotypic changes in transgenic *Arabidopsis thaliana* overexpressing *JcBSP1* and found that transgenic plants exhibited phenotypes of enlarged rosette leaves, flowers, and seeds. These findings laid the foundation for further research on the function of *BSP* genes in the plant growth and development.

## Materials & Methods

### Plant materials and nitrogen treatment

Four-year-old adult *J. curcas* were grown in the experimental field of Xishuangbanna Tropical Botanical Garden (21°54′N, 101°46′E; 580 m in altitude) in Yunnan Province. Wild-type *Arabidopsis thaliana* ecotype Columbia (Col-0) and the transgenic lines were grown in an environmentally controlled room at 22 °C under a 16-h light/8-h dark photoperiod. Wild-type *J. curcas* seeds were sown in sand that had been washed several times with distilled water and grown at 30 °C under a 12-h light/12-h dark photoperiod. Then, two-month-old *J. curcas* seedlings were randomly divided into three groups, with 12 plants per group. The three groups were treated with different concentrations of NH_4_NO_3_ solution (0, 5, and 50 mM). The seedlings were watered every week with 100 mL of a NH_4_NO_3_ solution per cup of seedlings.

### Sequence analysis

We used BLAST (http://www.ncbi.nlm.nih.gov/BLAST/) to analyze the cDNA sequence, CDS and amino acid sequence of *JcBSP* gene family members in the NCBI database. The conserved domains of deduced protein sequences were analyzed by the NCBI Conserved Domain Database (NCBI-CDD, https://www.ncbi.nlm.nih.gov/Structure/cdd/wrpsb.cgi). The alignment of amino acid sequences was performed using DNAMAN software (version 6, Lynnon Biosoft Corporation, Canada, https://www.lynnon.com/dnaman.html).

### Phylogenetic analysis

To examine the phylogenetic relationships of the BSP homologues from different species, we retrieved the deduced protein sequences from the NCBI database (https://www.ncbi.nlm.nih.gov/) and selected the sequences from species belonging to Euphorbiaceae and *Populus* with the highest sequence similarity to JcBSPs. Sequences of PtdPNI288 and PtdWIN4 were derived from the reference literature (Cooke & Weih, 2005; Wildhagen et al., 2010). A phylogenetic tree was built in the MEGA program (version 7.0, https://megasoftware.net/) using the neighbor-joining method with 1000 bootstrap replicates.

### RNA extraction

The samples for RNA extraction included different tissues (roots, stems, shoot apices, young leaves, mature leaves, male flowers, female flowers, fruits and seeds) of four-year-old adult *J. curcas*, stems collected from October 2019 to August 2020, two-month-old *J. curcas* seedlings treated with different concentrations of NH_4_NO_3_ solution for 0, 2, 4, 6, and 8 weeks, and leaves of one-month-old WT and transgenic *Arabidopsis*. These samples were quickly frozen in liquid nitrogen and stored at −80 °C. Total RNA was extracted using the silica adsorption method (Ding et al., 2008) , and the concentration and purity of RNA were detected by spectrophotometry and agarose gel electrophoresis, respectively.

### qRT-PCR analysis

Reverse transcription of total RNA was performed using the PrimeScript^®^ RT reagent kit with gDNA Eraser (Takara, Dalian, China). qRT-PCR was performed on the Roche 480 real-time PCR detection system using LightCycler^®^ 480 SYBR Green I Master Mix (Roche Diagnostics, Indianapolis, IN, USA). The qRT-PCR reactions were performed under the following conditions: 5 min at 95 °C for the initial denaturation, followed by 42 cycles of 10 s at 95 °C, 20 s at 57 °C, and 20 s at 72 °C for the PCR amplification, and 1 cycle of 30 s at 95 °C, 30 s at 65 °C and 0.06 °C/s heating up to 95 °C for the melting curve. Data was analyzed using the 2^−ΔΔCT^ method as described by Livak & Schmittgen (2001). All expression data obtained in the qRT-PCR assay were normalized to the expression of *JcActin1* (Zhang et al., 2013) and *AtActin2*. The primers used for qRT-PCR are listed in Table S1.

### Protein extraction

Stem samples of four-year-old adult *J. curcas* collected from October 2019 to August 2020 were also used for total protein extraction. The bark + phloem and xylem + pith dissected from stems were ground into powder mixtures. Fifty milligrams of powder were homogenized in 300 µl of protein extract buffer (50 mM Tris-HCl pH 7.4, 150 mM NaCl, 1 mM EDTA, 0.1% Triton X-100, 10% glycerol). The mixture was incubated at 4 °C for 30 min and then centrifuged at 12,000 rpm for 15 min at room temperature. The supernatant was collected and analyzed by a Bradford protein assay kit (BL524A, GUANGKE Technology Company, Kunming).

### Correlation analysis

Correlation analysis between the total protein concentration and *JcBSP1* expression was performed by the method of Spearman’s rank correlation using R package ggpubr (version 0.4.0, https://cran.r-project.org/).

### Construction of the *JcBSP1* overexpression vector and *Arabidopsis* transformation

The primers XD423 (CGAGCTCATGGCTATGGCGACGGTGAT) and XD424 (CGGATCCTCACTCTTCATCATAACGGA) carrying *Sac*I and *Bam*HI restriction sites, respectively, were used to clone the full-length *JcBSP1* CDS. Then, the PCR product was cloned into the pGEM-T Easy vector (Promega, Madison, WI, USA). To generate the *35S:JcBSP1* overexpression vector, *Sac*I and *Bam*HI were used to digest the plant transformation vector pOCA30 (Chen & Chen, 2002) and the pGEM-T Easy vector containing the *JcBSP1* sequence, respectively, and then, the resulting fragments were ligated by using T4 DNA Ligase (Promega). The generated *35S:JcBSP1* plasmid was transferred to *Agrobacterium tumefaciens* EHA105. Transformation of *Arabidopsis* was performed using the floral dip method (Clough & Bent, 1998).

## Results

### Identification of the members of the *JcBSP* gene family

**Figure 1:**
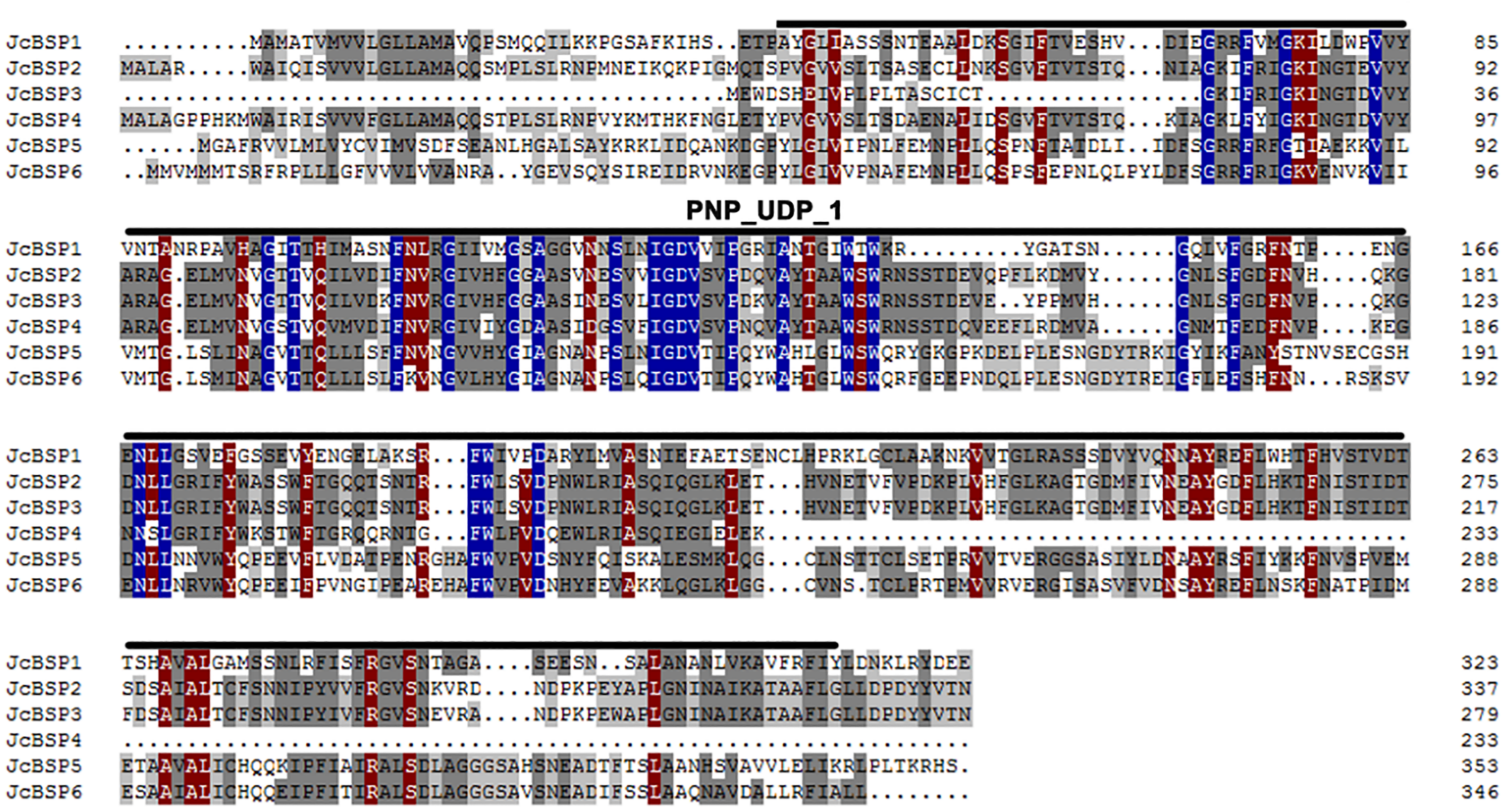
Protein sequence alignment of *JcBSP* family members of *J. curcas*. Identically conserved amino acid sequences are shown with a dark blue background, and partially conserved amino acid sequences are shown with grey and brown backgrounds; the conserved PNP_UDP_1 domain of JcBSP is indicated with overlining.

We found six members of the *JcBSP* gene family in *J. curcas* from the NCBI database using BLAST and designated them as *JcBSP1*, *JcBSP2*, *JcBSP3*, *JcBSP4*, *JcBSP5*, and *JcBSP6* (Table 1). All the *JcBSP* gene family members contain a conserved domain, purine nucleoside phosphorylase_uridine phosphorylase_1 (PNP_UDP_1), which is a signature of the phosphorylase superfamily (Fig. 1).

**Table 1:** Sequence information for members of the *JcBSP* family in *J. curcas*.

Gene name	GenBank accession number	cDNA (bp)	CDS (bp)	Number of amino acids (aa)
*JcBSP1*	XM_012218517	1,248	972	323
*JcBSP2*	XM_012214829	1,144	1,017	338
*JcBSP3*	XM_012218526	959	843	280
*JcBSP4*	XM_012218520	1,038	702	233
*JcBSP5*	XM_012222025	1,274	1,062	353
*JcBSP6*	XM_012222124	1,174	1,041	346

**Figure 2:**
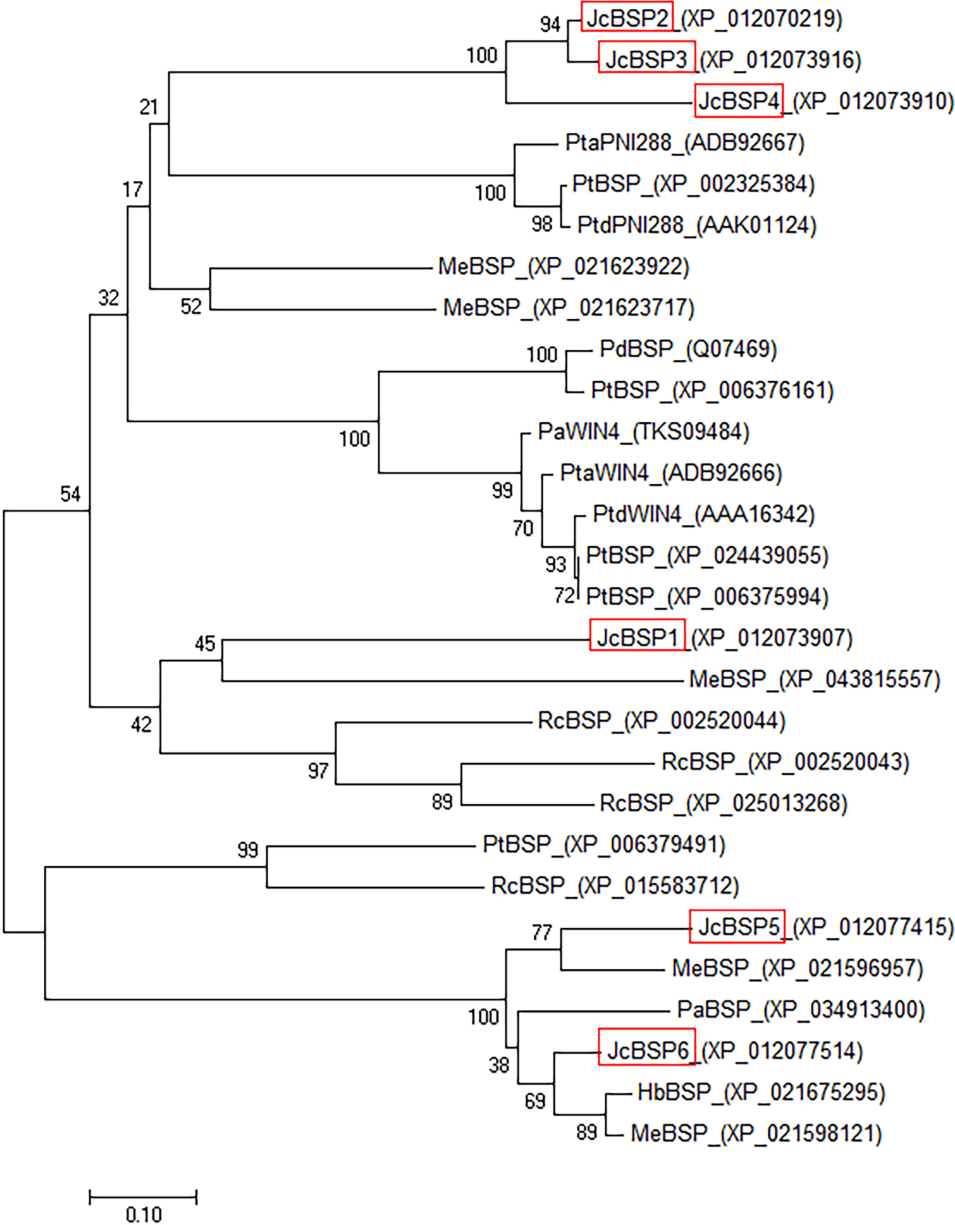
Phylogenetic tree analysis of BSP homologues. The homologues compared with *J. curcas* BSPs include *Ricinus communis* RcBSP; *Populus trichocarpa* PtBSP; *Populus deltoids* PdBSP; *Populus alba* PaBSP and PaWIN4; *Hevea brasiliensis* HbBSP; *Manihot esculenta* MeBSP; *P. trichocarpa* ×* P. deltoids* PtdWIN4 and PtdPNI288; and *P. trichocarpa* ×* P. alba* PtaWIN4 and PtaPNI288. The phylogenetic tree was constructed by the neighbor-joining method in MEGA 7.0 software; one thousand replicates were used for the bootstrap test; red frame: JcBSP family members.

To investigate the evolutionary relationships among *BSP* homologous genes, we performed phylogenetic analysis of genes from *J. curcas* and other species. The phylogenetic tree showed that JcBSP1, JcBSP5, and JcBSP6 were closely related to the BSP homologues from Euphorbiaceous species, and JcBSP2, JcBSP3, and JcBSP4 were closely related to the BSP homologues from poplar (Fig. 2).

### The expression patterns of *JcBSP*s in *J. curcas*

**Figure 3:**
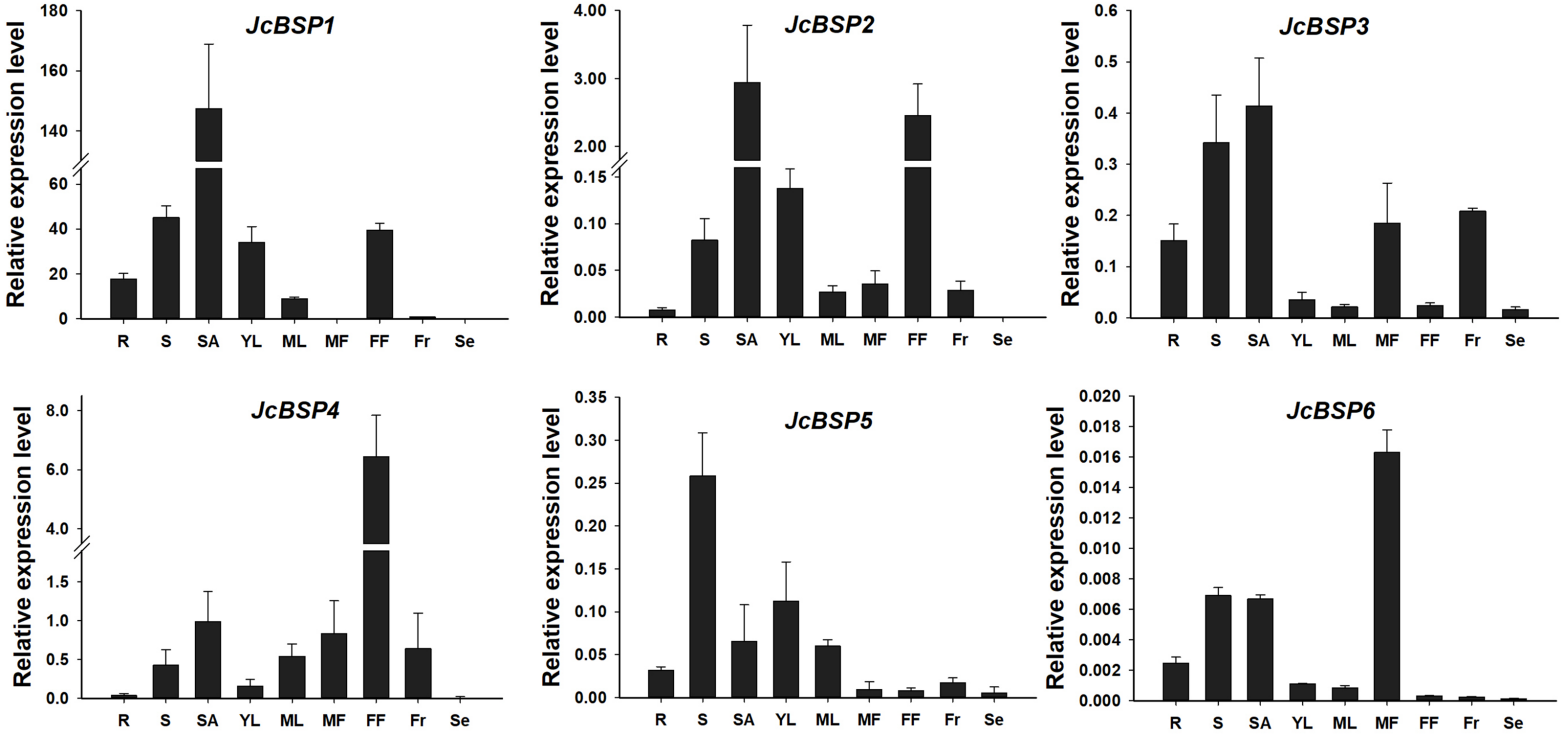
Expression analysis of *JcBSP*s in various tissues of adult *J. curcas*. The qRT-PCR results were obtained from three biological replicates and three technical replicates. The values were normalized to the expression of *JcActin1*. Error bars denote the standard deviation (SD) calculated from three biological replicates. R, roots; S, stems; SA, shoot apices; YL, young leaves; ML, mature leaves; MF, male flowers; FF, female flowers; Fr, fruits; Se, seeds.

To analyze the expression patterns of *JcBSP* family members, we used qRT-PCR to detect the expression levels of *JcBSP*s in roots, stems, shoot apices, young leaves, mature leaves, male flowers, female flowers, fruits, and seeds of adult *J. curcas*. The results showed that *JcBSP1* and *JcBSP2* exhibited similar expression patterns, in which both were highly expressed in shoot apices, stems, young leaves and female flowers; *JcBSP3* was mainly expressed in roots, stems, shoot apices, male flowers and fruits; the expression of *JcBSP4* was concentrated in reproductive tissues, with the highest expression level in female flowers; the expression of *JcBSP5* was concentrated in vegetative tissues, with the highest expression level in stems; and *JcBSP6* was remarkably expressed in male flowers (Fig. 3). Based on these results, we hypothesized that *JcBSP*s could play important roles in the growth and development of various organs, except seeds, in which all the members were barely expressed. This finding also indicates that JcBSPs may be vegetative storage proteins rather than seed storage proteins.

### Seasonal changes in total protein concentrations and *JcBSP* expression in the stems of *J. curcas*

In perennial deciduous trees, most nitrogen resources in senescing leaves are transported to perennial tissues (bark and wood), and stored as proteins during autumn and winter; the next spring, these proteins are hydrolyzed to amino acids, which are transported from perennial tissues to growing tissues (Chapin & Kedrowski, 1983; Cooke & Weih, 2005; Sauter, Cleve & Wellenkamp, 1989). Therefore, we investigated whether the total protein concentration in *J. curcas* stems was relevant to seasons.

**Figure 4:**
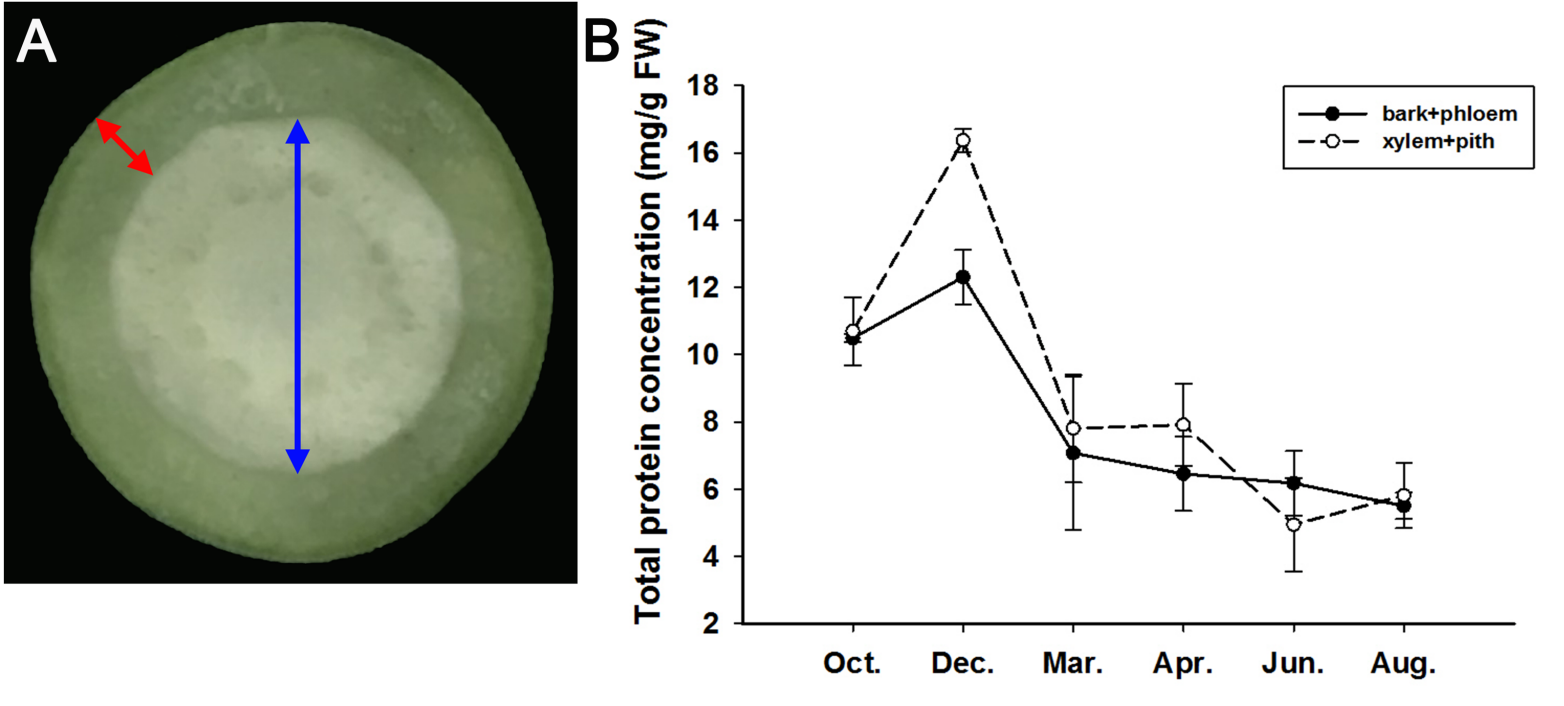
Seasonal changes in total protein concentrations in the stems of adult *J. curcas*. (A) Cross section of stems. The red arrow shows the bark and phloem, and the blue arrow shows the xylem and pith. (B) Total protein concentrations of *J. curcas* stems. The results were obtained from three biological replicates. Error bars denote the SD calculated from three biological replicates.

From October 2019 to August 2020, we sampled the stems of adult *J. curcas* in two parts (bark + phloem and xylem + pith), as shown in Fig. 4A, and examined the total protein concentrations of the samples. The results showed that the total protein concentrations in the bark + phloem and xylem + pith were approximately 10.5 mg/g FW in October; then, *J. curcas* entered the dormant stage in December, and the total protein concentrations reached a peak in the bark + phloem (12.3 mg/g FW) and xylem + pith (16.4 mg/g FW). When *J. curcas* began to enter the growing season in March, the total protein concentrations decreased sharply to 7 mg/g FW in the bark + phloem, which further decreased to 5.5 mg/g FW in August. The total protein concentration in the xylem + pith showed a similar trend (Fig. 4B). The total protein concentration in the *J. curcas* stem exhibited a seasonal change, which accumulated in autumn and winter and decreased in spring and summer. This result indicates that the total protein in the stems is a form of nitrogen storage during the overwintering period of *J. curcas*, and this protein is reallocated during the growing seasons.

**Figure 5:**
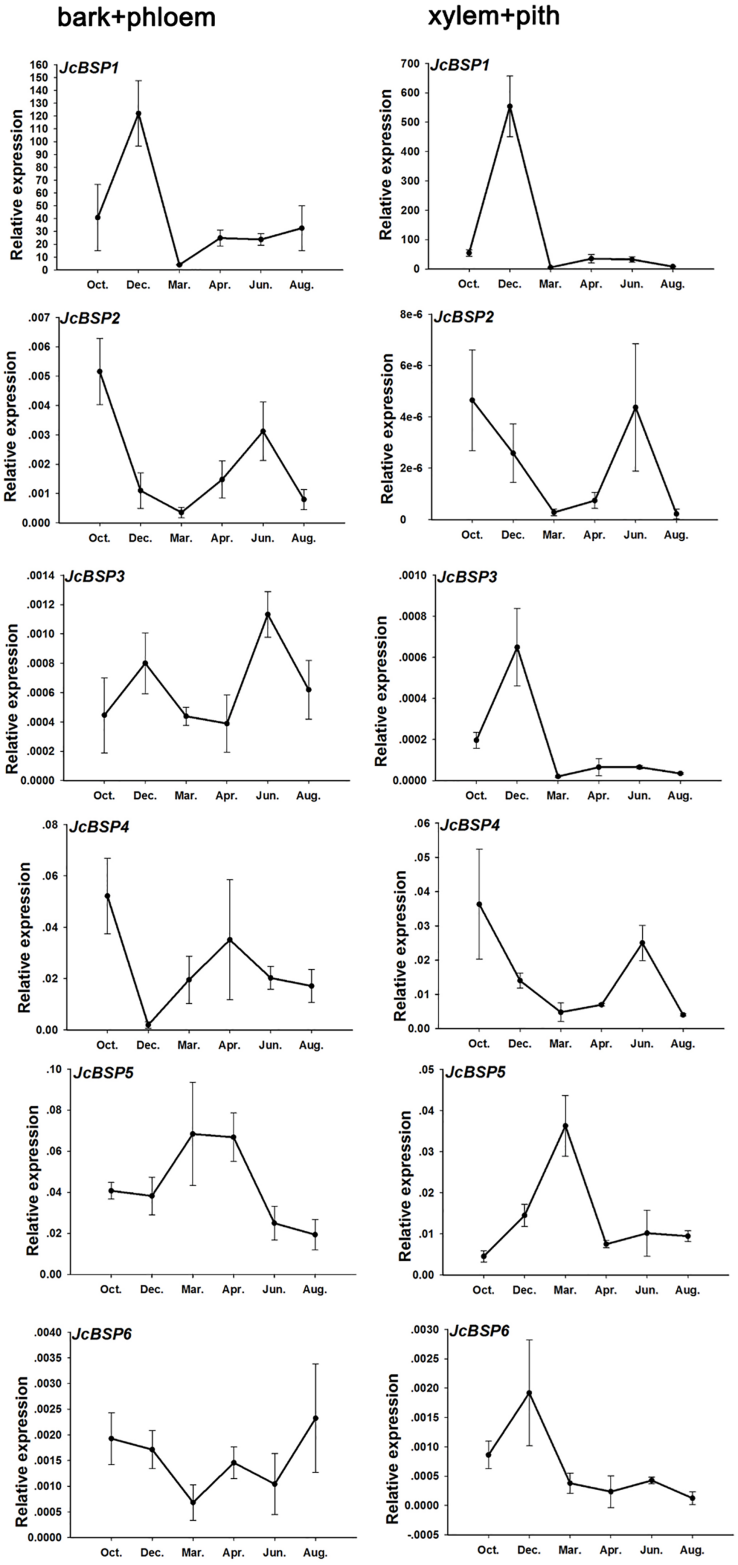
Seasonal changes in *JcBSP* expression in the two parts of *J. curcas* stems. The qRT-PCR results were obtained from three biological replicates and three technical replicates. The values were normalized to the expression of *JcActin1*. Error bars denote the SD calculated from three biological replicates.

**Figure 6:**
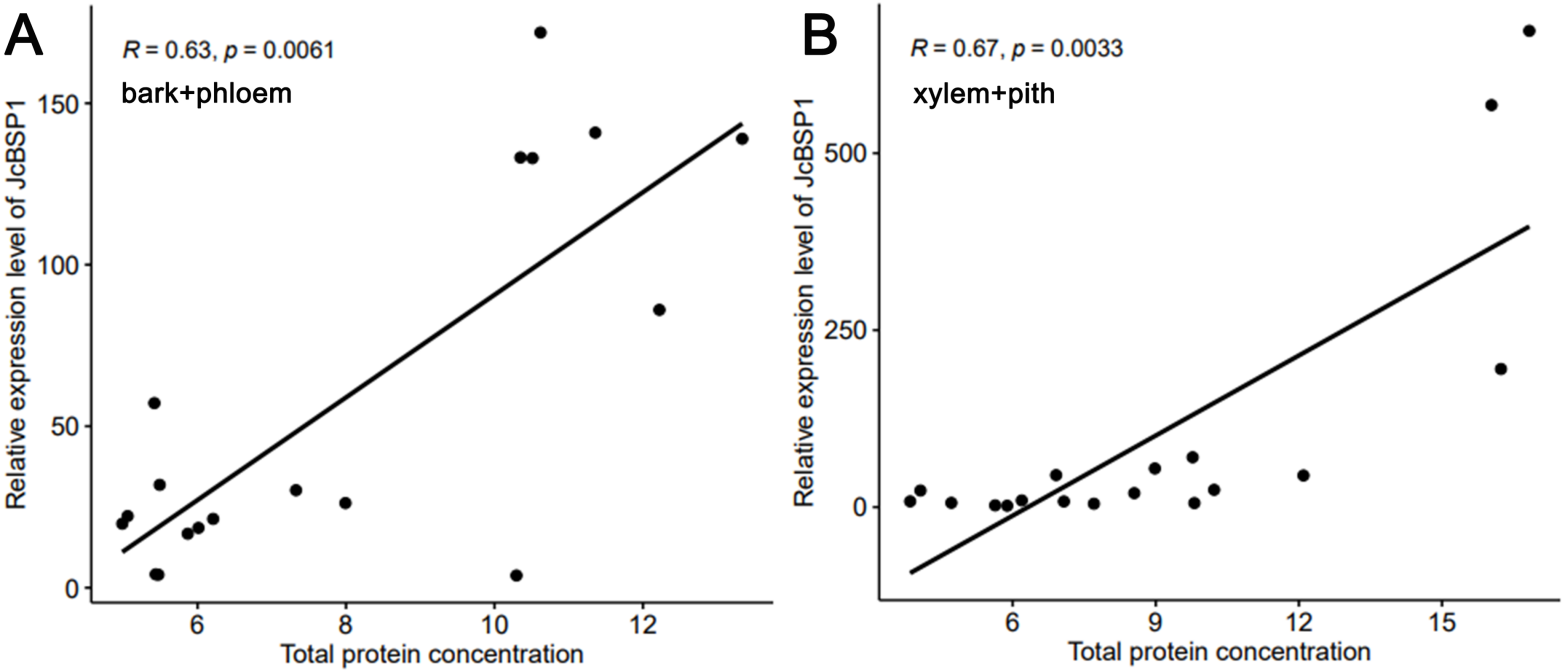
Correlation analysis between the seasonal changes in total protein concentration and *JcBSP1* expression in the bark + phloem (A) and the xylem + pith (B).

Based on the above results, we examined the seasonal course of *JcBSP* expression in the same samples mentioned above (Fig. 4A) to investigate whether the expression of *JcBSP*s shows the same seasonal changes as the total protein concentration. The results showed that the expression of *JcBSP* family members in stems exhibited different patterns over the seasonal course (Fig. 5). From autumn to winter, the expression of *JcBSP1* in the two parts of the stems increased rapidly, with a higher level in xylem + pith, and then decreased sharply in spring and remained low until August. The seasonal changes in *JcBSP1* expression in the two parts of the stem were entirely consistent with those of the total protein concentration. However, the seasonal expression patterns of *JcBSP2*, *JcBSP4* and *JcBSP5* in stems were inconsistent with those of the total protein concentration. In addition, only in the xylem + pith did the expression of *JcBSP3* and *JcBSP6* show the same seasonal changes as that of the total protein concentration, but their expression levels were much lower than that of *JcBSP1*. Therefore, we analyzed the correlation between seasonal changes in the total protein concentration and *JcBSP1* expression. It turned out that there were significant correlations between them in the bark + phloem (*r* = 0.63, *P* < 0.01) and the xylem + pith (*r* = 0.67, *P* < 0.01) (Fig. 6). These results suggest that *JcBSP1* might play an important role in seasonal nitrogen cycling.

### *JcBSP* expression in response to nitrogen

**Figure 7:**
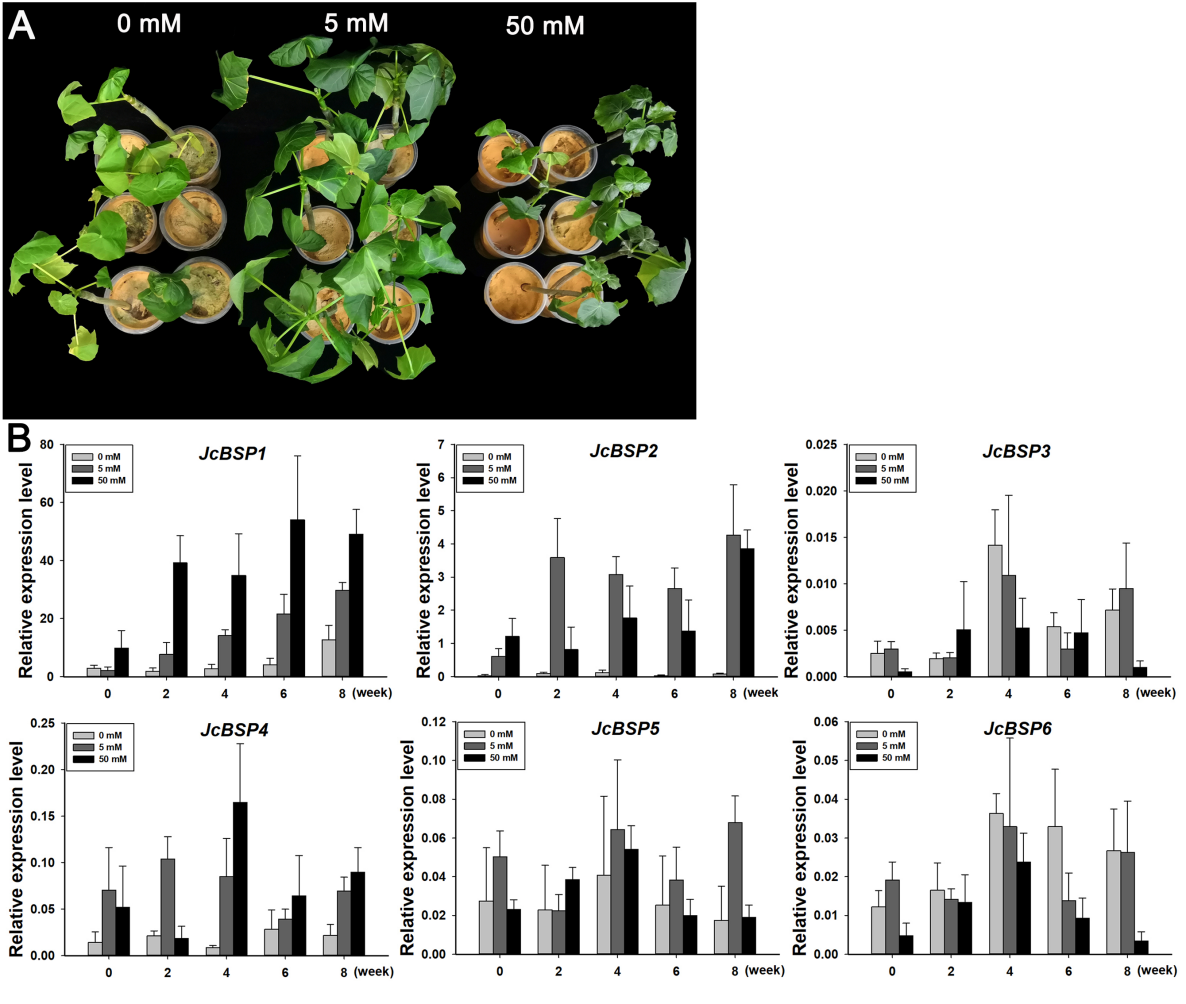
Changes in *JcBSP* expression in the leaves of two-month-old *J. curcas* seedlings treated with NH_4_NO_3_. (A) *J. curcas* seedlings treated with different concentrations of NH_4_NO_3_ for 8 weeks. (B)*JcBSP* expression in response to NH_4_NO_3_ treatment. The qRT-PCR results were obtained from three biological replicates and three technical replicates. The values were normalized to the expression of *JcActin1*. Error bars denote the SD calculated from three biological replicates.

To further verify the correlation between *JcBSP*s and seasonal nitrogen cycling, we investigated the response of these genes to nitrogen induction. We applied a 0, 5, and 50 mM NH_4_NO_3_ solution to two-month-old *J. curcas* seedlings. After 8 weeks of treatment, we found that the group treated with the 5 mM NH_4_NO_3_ solution grew better than the other two groups (Fig. 7A). This result indicated that nitrogen supply in a certain concentration range could effectively promote the growth of *J. curcas*.

We collected the leaves of *J. curcas* seedlings treated with different concentrations of NH_4_NO_3_ for 0, 2, 4, 6, and 8 weeks to detect changes in *JcBSP* family member expression. The results showed that the expression of *JcBSP1* increased obviously along with the increased NH_4_NO_3_ concentration and application duration; *JcBSP2* expression increased remarkably with the 5 mM NH_4_NO_3_ treatment for 2–8 weeks and with 50 mM NH_4_NO_3_ treatments for 8 weeks; *JcBSP4* expression was induced obviously with only the 50 mM NH_4_NO_3_ treatment for 4 weeks; however, the expression of *JcBSP3*, *JcBSP5* and *JcBSP6* was not induced by NH_4_NO_3_ treatment (Fig. 7B). The results indicated that *JcBSP1* and *JcBSP2* expression is responsive to nitrogen induction. In particular, *JcBSP1* expression was positively correlated with the nitrogen concentration and application duration. Combined with the seasonal changes in *JcBSP1* expression in stems, we further concluded that JcBSP1 might be a form of nitrogen storage in the seasonal nitrogen cycling in *J. curcas*.

### Overexpression of *J. curcas JcBSP1* in *Arabidopsis* led to enlarged rosette leaves, flowers, and seeds

**Figure 8:**
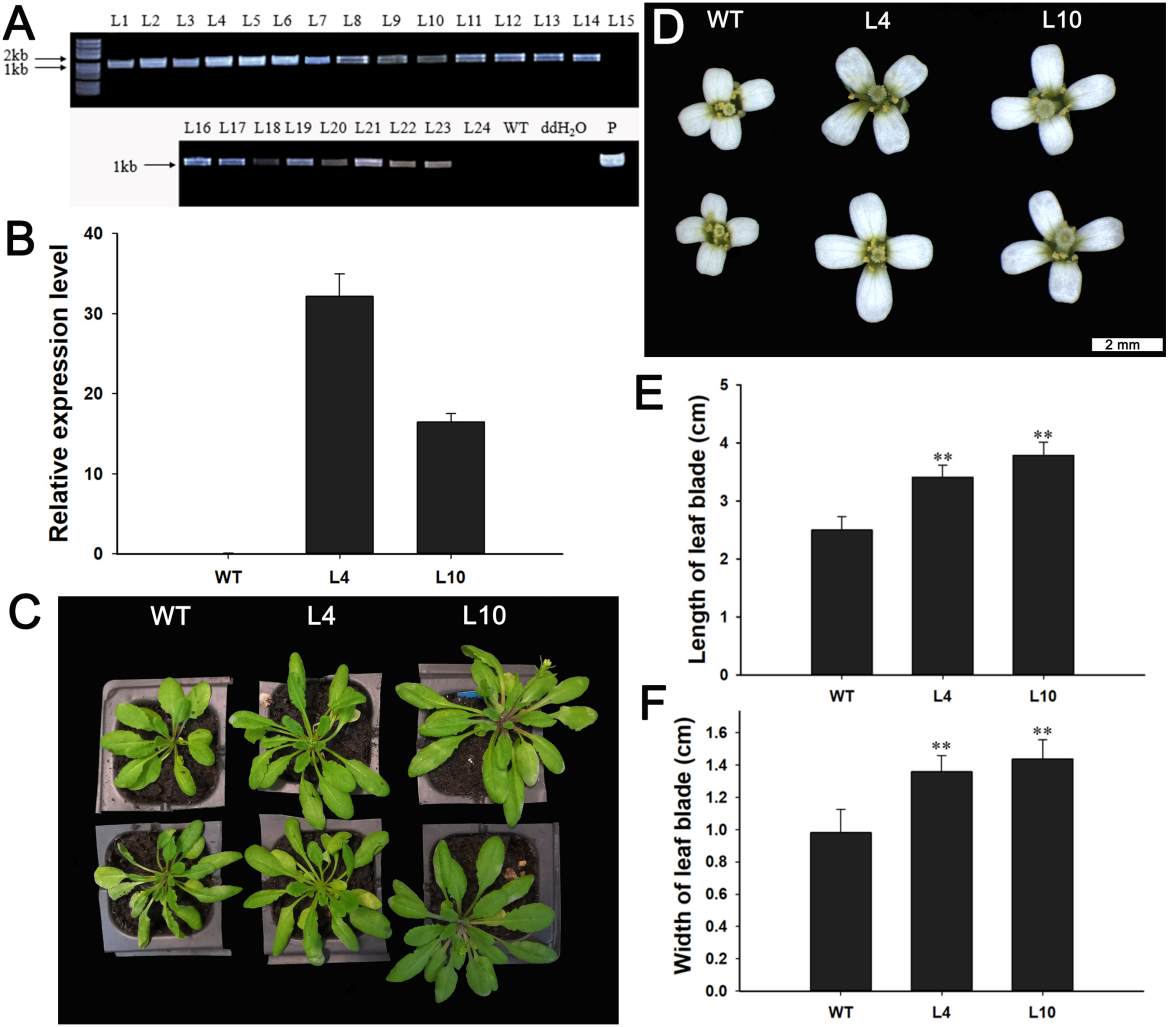
Phenotypic changes in *35S:JcBSP1* transgenic *Arabidopsis*. (A) PCR identification of *35S:JcBSP1* transgenic *Arabidopsis*. WT, wild-type negative control; ddH_2_O, ddH_2_O negative control; P, positive control. (B) qRT-PCR analysis of *JcBSP1* expression in WT and transgenic *Arabidopsis* (L4 and L10). The qRT-PCR results were obtained from three biological replicates and three technical replicates. The values were normalized to the expression of *AtActin2*. Error bars denote the SD calculated from three biological replicates. (C and D) Rosette leaves and flowers from WT and transgenic *Arabidopsis*. (E and F) Leaf length and width of WT and transgenic *Arabidopsis*. The values are presented as the means ± standard deviations (*n* = 8). Student’s *t*-test was used for significance analysis: **P ≤ 0.01.

Next, we investigated the function of *JcBSP1* in plant growth and development in transgenic *Arabidopsis*. Twenty-two independent *35S:JcBSP1* transgenic *Arabidopsis* lines were generated (Fig. 8A). *JcBSP1* expression in seven transgenic lines showing similar phenotypes was analyzed, and most of transgenic lines yielded abundant transgene expression (Fig. S1). We investigated in further detail the phenotypes of two independent transgenic lines, L4 and L10, which exhibited high and intermediate expression levels of *JcBSP1*, respectively (Fig. 8B).

**Figure 9:**
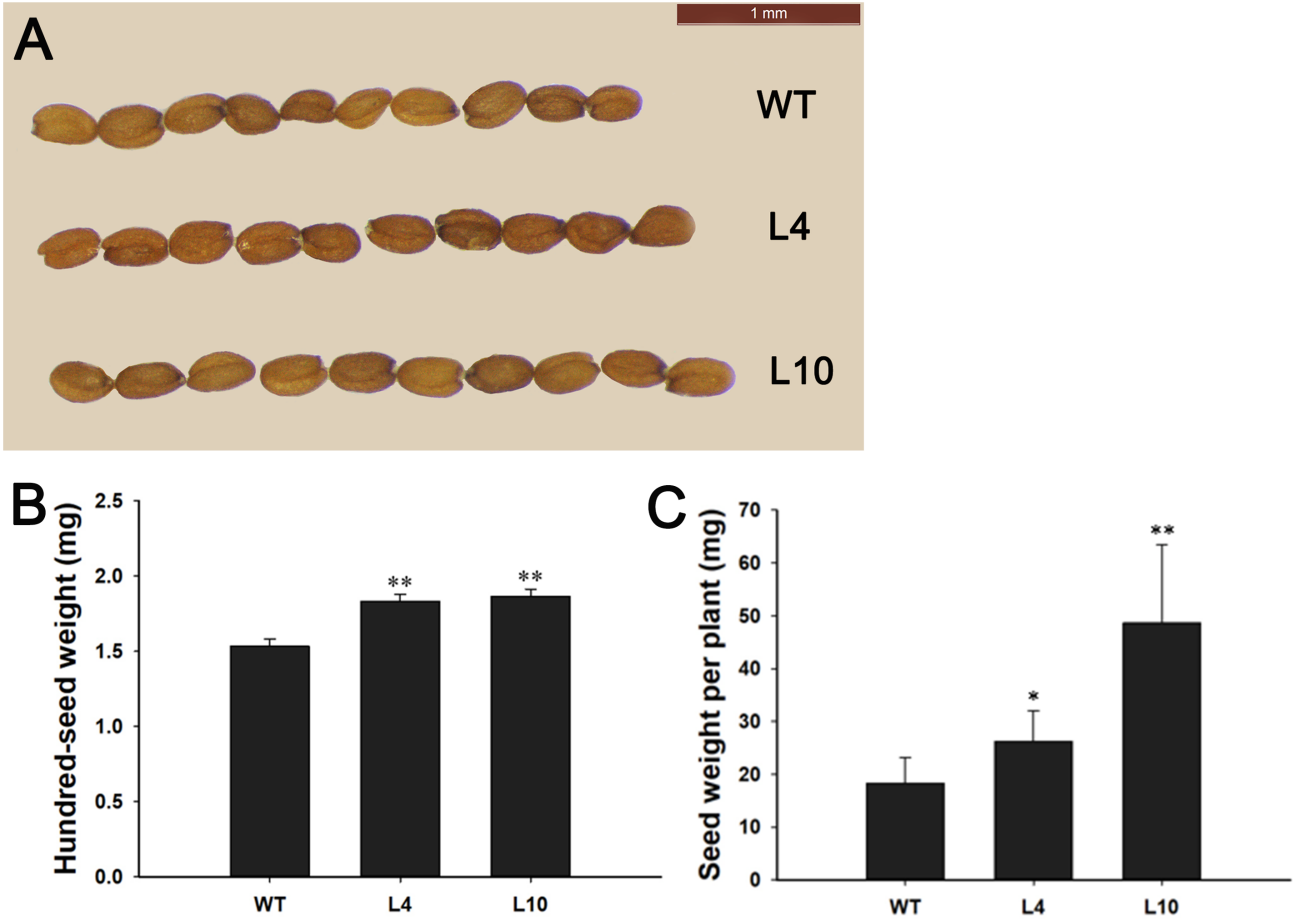
Overexpression of *JcBSP1* increased the size, weight, and yield of seeds in transgenic *Arabidopsis*. Seed size (A), hundred-seed weight (B) (*n* = 3) and seed yield per plant (C) (*n* = 6) were analyzed in WT and transgenic *Arabidopsis* lines L4 and L10. The values are presented as the means ± standard deviations. Student’s *t*-test was used for significance analysis: *P ≤ 0.05, **P ≤ 0.01.

During plant growth and development, we found that *35S:JcBSP1* transgenic *Arabidopsis* produced larger rosette leaves and flowers than the wild-type (WT) plants (Figs. 8C and 8D). As shown in Figs. 8E and 8F, the rosette leaf lengths and widths were all significantly increased in transgenic lines. And larger seeds and significantly increased hundred-seed weights were found in transgenic plants (Figs. 9A and 9B). Consequently, the seed yields in transgenic lines were significantly higher than that of the WT (Fig. 9C). These results indicated that *JcBSP1* was able to affect plant growth and development.

## Discussion

BSP, a kind of VSP, is a main nutrient storage protein in perennial woody plants and a form of nitrogen storage in vegetative tissues (Cooke & Weih, 2005; Staswick, 1994). It is different from seed storage protein, which accumulates during seed maturation and provides a nitrogen source for embryo development (Autran, Halford & Shewry, 2001; Gacek, Bartkowiak-Broda & Batley, 2018; Kawakatsu et al., 2010). In *Populus*, *BSP* has been found to be highly expressed in the bark, dormant cambium and bud (Coleman, Baíiados & Chen, 1994; Cooke & Weih, 2005) , and the *bspA* promoter has been shown to be predominantly active in bark (Zhu & Coleman, 2001). In this study, we identified six members of the *JcBSP* gene family in *J. curcas*, and none of them were expressed in seeds (Fig. 3), indicating that JcBSP might be a nutrient storage protein rather than seed storage protein. In addition, *JcBSP1*, *JcBSP2* and *JcBSP4* were highly expressed in female flowers, and *JcBSP3* and *JcBSP6* were relatively highly expressed in male flowers, suggesting that they may be involved in the development of female and male flowers, respectively.

In perennial woody plants, BSP plays an important role in seasonal nitrogen cycling (Wetzel, Demmers & Greenwood, 1989b; Wetzel & Greenwood, 1989; Wildhagen et al., 2010). During autumn and winter, nitrogen-rich amino acids are transported from senescing leaves to perennial tissues and subsequently used to synthesize proteins for nitrogen storage (Geßler, Kopriva & Rennenberg, 2004; Hörtensteiner & Feller, 2002). BSP is the main form of nitrogen storage in trees during the dormant period, which accumulates in autumn and winter (Cooke & Weih, 2005; Wetzel, Demmers & Greenwood, 1989b). In this study, we found that the seasonal changes in *JcBSP1* expression in stems were consistent with those of the total protein concentration, as both increased in autumn and winter and then decreased in spring and summer (Figs. 4B and 5). And there is a significant correlation between seasonal changes in the total protein concentration and *JcBSP1* expression (Fig. 6), suggesting that JcBSP1 might be the main protein stored in the stem of *J. curcas* during overwintering. Moreover, the expression of *JcBSP1* was positively correlated with the nitrogen concentration and application duration (Fig. 7B). Therefore, we hypothesized that JcBSP1 might play an important role in the seasonal nitrogen cycling of *J. curcas*, acting as a form of nitrogen storage in the stems during overwintering. In addition, this study was conducted in the Xishuangbanna Tropical Botanical Garden, Chinese Academy of Sciences, which is located in a tropical region of China. According to rainfall, there are two seasons, a rainy season from May to October and a dry season from November to April of the following year, in Xishuangbanna area. The dry season is further divided into the foggy-cool season from November to February of the following year and the dry-hot season from March to April. Although the foggy-cool season has little precipitation, there is a large amount of dense fog from night to noon, which has a certain compensation effect on the water demand of plants in dry season; the dry-hot season has a dry climate, low precipitation and large daily temperature differences (Zhao et al., 2009). As shown in Figs. 4B and 5, both of the total protein concentration and *JcBSP1* expression in the stems were decreased to a very low level at the beginning of the dry-hot season. Hence, further studies are required to link the seasonal changes in total protein concentration and *JcBSP1* expression to possible drought-related protein mobilization.

It is well known that nitrogen is an important nutrient for plant growth and development. Lemaitre et al. (2008) showed that when *Arabidopsis* grew under low nitrogen conditions, rosette biomass and seed yield were limited. Storage proteins are considered as nitrogen source that are utilized for plant growth (Sözen, 2004; Titus & Kang, 1982). In this study, we found that overexpression of *JcBSP1* could promote the growth and development of rosette leaves, flowers, and seeds in transgenic *Arabidopsis* (Figs. 8 and 9). This finding further indicates the JcBSP1 might be a form of nitrogen storage in plants, serving as a nutrient provider. Similarly, overexpression of a storage protein gene *AmA1* in potato could increase the growth and production of tubers (Chakraborty, Chakraborty & Datta, 2000). By analyzing cell architectures, the cell areas in cortex, perimedullary and pith regions of the tuber were found to be increased, which indicated the AmA1 storage protein in potato tuber was correlated with cell growth (Agrawal et al., 2013). In cabbage, when the nitrogen supply can’t meet the need of plant growth, the leaf cells became smaller while the number of cell layers remained unchanged (Kano et al., 2007). It turns out that both endogenous and exogenous nitrogen sources could affect cell growth. Accordingly, overexpression of *JcBSP1* in transgenic *Arabidopsis* may also promote the cell growth, resulting in enhanced plant growth and production. Furthermore, it is worthy to mention here that although the expression level of *JcBSP1* in the transgenic line L4 was higher than that in L10 (Fig. 8B), the leaves and seeds in L4 were relatively smaller than those in L10 (Figs. 8C, 8E, 8F; 9). We hypothesized that this phenotype might be caused by the excessively high *JcBSP1* transgene expression, which might lead to excess JcBSP1 protein storage and subsequently excess nitrogen accumulation in L4 plants. Previous study showed that under the excess nitrogen conditions, both cell number and size were found to be reduced in leaves (MacAdam, Volenec & Nelson, 1988). In addition, about half of the Rubisco are inactive or only half of the catalytic sites are functional, which certainly leads to a decrease in photosynthetic efficiency and therefore a retardation in plant growth (Chapin, Schulze & Mooney, 1990; Cheng & Fuchigami, 2000; Millard, 1988). As shown in Fig. 7A, the excessive nitrogen supply does have a certain negative impact on *J. curcas* growth. Consistently, Barbosa et al. (2010) also found that when the adding nitrogen concentration was below 40 mM, it stimulated *Arabidopsis* root growth, while the concentration was higher than 40 mM, root elongation was inhibited.

In addition, VSPs may also play a role in plant defense. In *Arabidopsis*, *AtVSP1* and *AtVSP2* have been shown to enhance plant resistance to diseases and insects (Berger, Mitchell-Oldsb & Stotz, 2002; Ellis & Turner, 2001; Liu et al., 2005). Furthermore, *AtVSP1* and *AtVSP2* have been found to be highly expressed in flowers (Utsugi et al., 1998), which implies a mechanism used by *Arabidopsis* to protect reproductive structures (Liu et al., 2005). Interestingly, most *JcBSP*s were also highly expressed in female or male flowers (Fig. 3). Thus, in addition to being a provider of nitrogen resources, *JcBSP*s may also play other roles in plant growth and development, which requires further study.

## Conclusions

In this study, six members of the *JcBSP* gene family were identified in *J. curcas*, which were expressed in various tissues, except seeds. Among these members, only the expression of *JcBSP1* was positively correlated with the total protein concentration in the stems during seasonal changes and with the exogenous nitrogen application. We thus supposed that JcBSP1 could play an important role in seasonal nitrogen cycling as a form of nitrogen storage. By the function analysis of *JcBSP1* in transgenic *Arabidopsis*, we found that *JcBSP1* was able to enhance the plant growth and production. This suggests that *JcBSP1* could be useful in crop breeding.

## Supplemental Information

10.7717/peerj.12938/supp-1 Supplemental Information 1 QRT-PCR primers list

10.7717/peerj.12938/supp-2 Supplemental Information 2 *JcBSP1* expression in wild-type (WT) and transgenic *Arabidopsis* lines The qRT-PCR results were obtained from three biological replicates and three technical replicates. The values were normalized to the expression of *AtActin2*. Error bars denote the SD from three biological replicates.

10.7717/peerj.12938/supp-3 Supplemental Information 3 Seasonal changes in JcBSP expression in the stems

10.7717/peerj.12938/supp-4 Supplemental Information 4 JcBSP expression in response to nitrogen

10.7717/peerj.12938/supp-5 Supplemental Information 5 JcBSPs expression patterns

10.7717/peerj.12938/supp-6 Supplemental Information 6 Seasonal changes in total protein concentrations in the stems

10.7717/peerj.12938/supp-7 Supplemental Information 7 Leaf length and width

10.7717/peerj.12938/supp-8 Supplemental Information 8 Hundred-seed weight

10.7717/peerj.12938/supp-9 Supplemental Information 9 Seed yield per plant

## Additional Information and Declarations

### Data Availability

The following information was supplied regarding data availability: The qRT-PCR primers, *JcBSP1* expression in wild-type (WT) and transgenic *Arabidopsis* lines and raw measurements are available in the Supplementary Files.
