# Supplementary material for: Characterization of the bark storage protein gene (JcBSP) family in the perennial woody plant Jatropha curcas and the function of JcBSP1 in Arabidopsis thaliana
Source: PeerJ. 2022 Feb 8;10:e12938. doi: 10.7717/peerj.12938 (PMC8833228; doi:10.7717/peerj.12938)
Supplement: Supplemental Information 1 [file peerj-10-12938-s001.docx]

**Table S1** **qRT-PCR primers list**

| Gene | Forward (5' - 3') | Reverse (5' - 3') |
| --- | --- | --- |
| *JcActin1* | CTCCTCTCAACCCCAAAGCCAA | CACCAGAATCCAGCACGATACCA |
| *AtActin2* | TGTGCCAATCTACGAGGGTTT | TTTCCCGCTCTGCTGTTGT |
| *JcBSP1* (in *Arabidopsis*) | GGTTGTGACTGGACTGAGGG | GTTGCTCGACATAGCTCCCA |
| *JcBSP1* | CCAGTAACATTGAGTTCGCAGAGAC | AGTCCAGTCACAACCTTGTTCTTT |
| *JcBSP2* | ATCAAATGGCTTTAGCAAGGTGG | TGAGTGGAGGTGACAGTGAAGACTC |
| *JcBSP3* | TTCAATTACATGGAATGGGATAGCC | TCCACAAGAATTTGCACAGTCGT |
| *JcBSP4* | AAGTGTGTTCATTGGTGATGTTTCC | CGTCCTAGTGAGTTATTTCCCTCCT |
| *JcBSP5* | ACATTTAGGTCTTTGGAGTTGGC | CACACTCACTCACATTCGTACTGTAA |
| *JcBSP6* | GAGTTGGCAGAGATTCGGAGAGG | TACAGGAAAGATCTCTTCAGGTTGGT |
