# Supplementary figures and images for: Characterization of the bark storage protein gene (JcBSP) family in the perennial woody plant Jatropha curcas and the function of JcBSP1 in Arabidopsis thaliana
Source: PeerJ. 2022 Feb 8;10:e12938. doi: 10.7717/peerj.12938 (PMC8833228; doi:10.7717/peerj.12938)

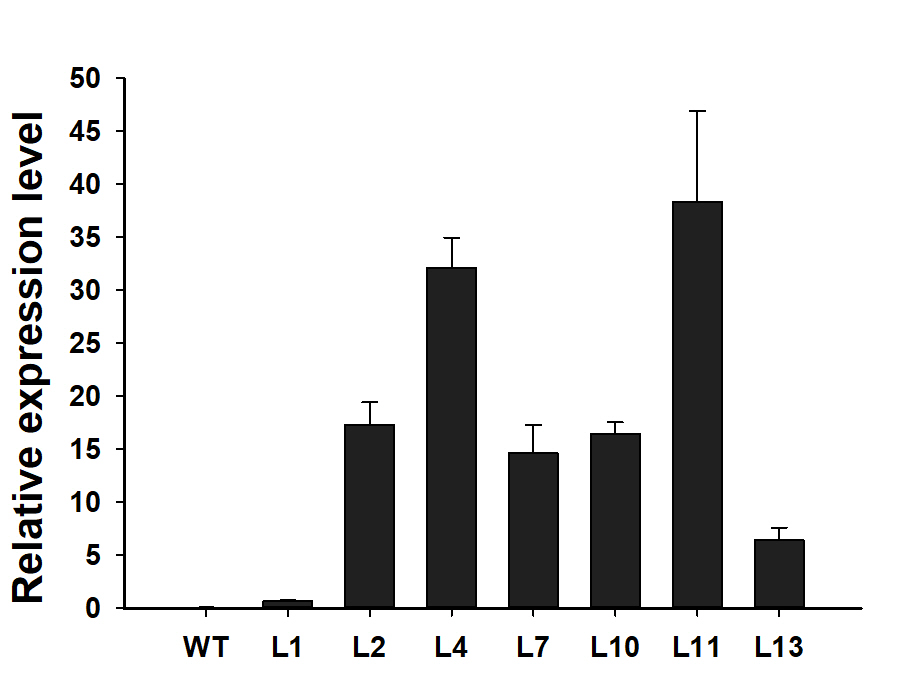

Supplement: Supplemental Information 2 — The qRT-PCR results were obtained from three biological replicates and three technical replicates. The values were normalized to the expression of AtActin2. Error bars denote the SD from three biological replicates. [file peerj-10-12938-s002.jpg]
